# Renal functional reserve predicts GFR response to empagliflozin in RENALIS and RACELINES clinical trials

**DOI:** 10.1093/ndt/gfag025

**Published:** 2026-02-10

**Authors:** Michaël J B van Baar, Petter Bjornstad, Daan J Touw, Jaap A Joles, Merle M Krebber, David Z I Cherney, Daniël H van Raalte, Marcel H A Muskiet

**Affiliations:** Diabetes Center, Department of Internal Medicine, Amsterdam UMC, Amsterdam, The Netherlands; Division of Endocrinology, Department of Pediatrics, and Division of Metabolism, Endocrinology and Nutrition, Department of Medicine, University of Washington School of Medicine, Seattle, WA, USA; Department of Clinical Pharmacy and Pharmacology, University of Groningen, UMCG, Groningen, The Netherlands; Department of Nephrology and Hypertension, UMC, Utrecht, The Netherlands; Department of Nephrology and Hypertension, UMC, Utrecht, The Netherlands; Department of Medicine, Division of Nephrology, University Health Network, Toronto General Hospital Research Institute, Toronto, Ontario, Canada; Diabetes Center, Department of Internal Medicine, Amsterdam UMC, Amsterdam, The Netherlands; Diabetes Center, Department of Internal Medicine, Amsterdam UMC, Amsterdam, The Netherlands; Department of Medicine, Division of Nephrology, University Health Network, Toronto General Hospital Research Institute, Toronto, Ontario, Canada; Department of Internal Medicine, Division of Endocrinology, Leiden University Medical Center, Leiden, The Netherlands

**Keywords:** DPP-4 inhibition, glomerular hyperfiltration, renal functional reserve, SGLT2-inhibition, type 2 diabetes

## Abstract

**Background:**

Glomerular hyperfiltration is common in type 2 diabetes (T2D) and may reflect reduced nephron number and/or perturbed intrarenal hemodynamics. Renal functional reserve (RFR), the capacity to increase glomerular filtration rate (GFR) with physiological stimuli (e.g. a meal), may help reveal single-nephron hyperfiltration in patients with preserved baseline whole-kidney GFR. We hypothesized that reduced postprandial RFR predicts the acute hemodynamic GFR response in T2D to the sodium-glucose cotransporter 2 (SGLT2) inhibitor empagliflozin, but not to the dipeptidyl-peptidase-4 inhibitor linagliptin or a sulfonylurea.

**Methods:**

This analysis pooled data from two 8-week randomized, double-blind, parallel-group mechanistic trials, encompassing 71 T2D patients with preserved whole-kidney GFR [mean ± standard deviation age 65 ± 7 years, boby mass index 30.4 ± 3.9 kg/m^2^, glycated hemoglobin A1c 7.8 ± 1.0% (61.6 ± 11.0 mmol/mol), GFR 86.5 ± 17.6 mL/min/1.73 m^2^]. Patients received empagliflozin (10 mg; *N* = 20), linagliptin (5 mg; *N* = 27) or sulfonylurea (glimepiride 1 mg or gliclazide 30 mg; *N* = 24), in addition to metformin. Measured (m)GFR and effective renal plasma flow (ERPF) were determined by inulin/iohexol and para-aminohippurate clearance, respectively, based on timed urine sampling in fasting and post-protein-rich meal conditions. Intrarenal hemodynamics were calculated using Gomez equations; fractional sodium excretion (FE_Na_) and systemic hemodynamics were evaluated.

**Results:**

The meal increased mGFR (+7.3 ± 1.7 mL/min/1.73 m^2^; *P* < .001) and ERPF (+44.3 ± 14.9 mL/min/1.73 m^2^; *P* = .005), with concomitant decrease in renal vascular resistance (RVR; −0.02 ± 0.01 mmHg/L/min; *P* < .001), driven by reduced afferent arteriolar resistance (−1068 ± 241dyne/s/cm^5^; *P* < .001) and lower FE_Na_ (−0.21 ± 0.05; *P* < .001). Postprandial mGFR changes did not correlate with baseline mGFR, but did correlate with postprandial RVR change (r: −0.57; *P* < .001). After 8 weeks, mGFR tended to decrease with sulfonylurea (*P* = .054) and decreased with empagliflozin (−9.1 ± 3.2 mL/min/1.73 m^2^; *P* = .016), but not with linagliptin. Baseline meal-induced mGFR changes correlated with 8-week treatment-induced mGFR changes with empagliflozin (r: 0.88; *P* < .001), but not with linagliptin or sulfonylurea.

**Conclusion:**

Baseline postprandial RFR predicts GFR-dipping with empagliflozin after 8 weeks, but not with GFR changes following linagliptin or sulfonylurea. As initial GFR dipping is associated with long-term kidney benefit, RFR warrants evaluation as a potential additional tool to personalize SGLT2 inhibitor therapy.

**Trial registration:**

ClinicalTrials.gov: NCT02106104 (RENALIS) and NCT03433248 (RACELINES).

## Graphical Abstract

**Graphical Abstract:**
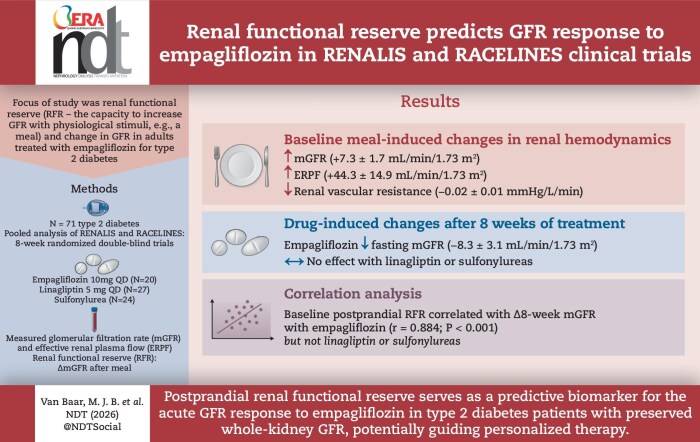

KEY LEARNING POINTS **What was known:**

- Glomerular hyperfiltration is common in type 2 diabetes, is linked to faster kidney function decline, and can exist subclinically at the single-nephron level when whole-kidney glomerular filtration rate (GFR) is within normal range.
- Renal functional reserve (RFR) testing may help reveal subclinical single-nephron hyperfiltration, yet its clinical utility as a biomarker remains uncertain.
- Sodium-glucose cotransporter 2 (SGLT2) inhibitors acutely lower GFR at treatment initiation through intrarenal hemodynamic mechanisms, and the magnitude of this initial “GFR dip” is linked to long-term kidney protection.

**This study adds:**

- RFR, measured after a high-protein meal, predicts the acute GFR response to SGLT2 inhibition; that is, a reduced RFR—indicative of subclinical single-nephron hyperfiltration—identifies patients who will experience the largest treatment-induced “GFR dip.”
- RFR may represent a dynamic biomarker that provides additional insight beyond baseline whole-kidney (estimated or measured) GFR and albuminuria, potentially helping to identify patients who could benefit from SGLT2 inhibition.

**Potential impact:**

- Incorporating RFR testing may help facilitate earlier identification of patients at risk for kidney function decline despite having normal baseline whole-kidney GFR.
- Pending appropriate validation and improvements in clinical feasibility, RFR assessment might have potential as a dynamic biomarker to inform individualized SGLT2 inhibitor therapy use in patients with type 2 diabetes without established chronic kidney disease.
- Future studies should evaluate whether RFR-guided treatment strategies have the potential to support more tailored clinical decision-making and improve long-term kidney outcomes in diabetes care.

## INTRODUCTION

Glomerular hyperfiltration, common in type 2 diabetes (T2D), results from obesity-/diabetes-induced changes in structural and dynamic factors that regulate glomerular filtration rate (GFR). Whole-kidney hyperfiltration (GFR >130–140 mL/min/1.73 m²) typically occurs before albuminuria onset and/or GFR decline [1]. Hyperfiltration predisposes to progressive nephron damage by increasing glomerular hydraulic pressure (P_GLO_) and promoting transcapillary convective flux of ultrafiltrate and macromolecules, including albumin. In chronic kidney disease or longstanding T2D, hyperfiltration in single remnant nephrons, to compensate for reduced nephron numbers, is believed to accelerate GFR decline [1].

A stress test designed to evaluate the full filtration capacity of the kidneys, known as renal functional reserve (RFR), using a high protein load or amino acids/dopamine infusion [1–4], may offer a method to detect single-nephron hyperfiltration in patients with a whole-kidney GFR within normal range. The assumption is that RFR is determined by the number of nephrons multiplied by the single nephron GFR. In individuals with impaired nephron endowment, single nephron GFR may already be near maximal to compensate and sustain overall filtration. As a result, RFR is likely to be blunted. This helps explain why preexisting elevations in P_GLO_ and effective renal plasma flow (ERPF) limit any further increase in GFR. However, the clinical utility of this method remains uncertain.

Sodium-glucose cotransporter 2 (SGLT2) inhibitors improve cardiovascular–kidney outcomes across diverse populations through several mechanisms [5], including improvements in renal hemodynamics, exemplified by acute GFR-dipping observed after drug initiation [1, 6, 7]. By blocking glucose/sodium (Na) reabsorption in the proximal tubule, they promote glycosuria and (transiently) raise fractional Na excretion (FE_Na_). This restores NaCl concentrations at the macula densa, activating tubuloglomerular feedback (TGF), which reduces (single nephron) hyperfiltration by increasing afferent renal arteriolar resistance (R_A_) in whole-kidney hyperfiltration [7], and reducing efferent renal arteriolar resistance (R_E_) in preserved/reduced GFR [6]. Additionally, increased intraluminal volume results in a retrograde rise in hydraulic pressure within Bowman’s space, which constrains filtration pressure and contributes to the mitigation of hyperfiltration [1].

Patients with baseline hyperfiltration show a clear kidney hemodynamic response to SGLT2 inhibition, evidenced by greater mGFR reductions in hyperfiltering versus normofiltering T1D patients [7]. Thus, since baseline hyperfiltration leads to larger initial GFR dips, which associates with long-term benefits [8–10], identifying hyperfiltering individuals, even with normal whole-kidney GFR (i.e. subclinical hyperfiltration), may have clinical implications. In this context, RFR may serve as a predictive marker of kidney-responsiveness to treatment. We hypothesized that a reduced RFR, potentially reflecting single-nephron hyperfiltration, could help identify patients most likely to experience more favorable mGFR responses to SGLT2-inhibition—characterized by an initial GFR decline followed by long term stabilization. This exploratory concept could inform future evaluation of RFR as a potential (dynamic) biomarker for personalizing therapy and optimizing kidney outcomes. Dipeptidyl-peptide-4 (DPP-4) inhibitors and sulfonylureas serve as controls, as they do not improve kidney outcomes beyond glucose lowering [11, 12], and lack drug-specific kidney hemodynamic effects [6, 13–16].

## MATERIALS AND METHODS

### Trial design and population

This analysis pools data from two 8-week, phase IV, randomized, double-blind, comparator-controlled, parallel-group, mechanistic intervention trials (RENALIS (RENoprotection in diAbetes by LInagliptin versus Sulfonylurea) [13, 14] and monotherapy-phase of RACELINES (Renal Actions of Combined Empagliflozin and LINagliptin in Type 2 diabetES) [15]) conducted at Amsterdam UMC, location VUMC, Amsterdam, the Netherlands. The local institutional review board, ethics committee and competent local authorities approved the trial protocols and its amendments. The trials complied with the Declaration of Helsinki and Good Clinical Practice, and are registered with ClinicalTrials.gov (ID: NCT02106104 and NCT03433248). Written informed consent was obtained from all patients prior to any trial-related activities.

Eligibility criteria in RENALIS and RACELINES were highly comparable and have been described previously [13–15]. In brief, all patients were Caucasian, men or postmenopausal women, aged 35–75 years, had T2D, received metformin monotherapy, had an glycated hemoglobin A1c (HbA1c) of 6.5%–9.5% (47.5–80 mmol/mol), and a BMI of ≥25 kg/m^2^. In cases of hypertension and/or albuminuria, treatment included a RAS blocker for 3 months or longer. The main exclusion criteria were history of pancreatic, active liver or malignant disease, an estimated GFR <45 mL/min/1.73 m^2^, urinary retention (confirmed via bladder ultrasound at screening), current urinary tract infection, or use of diuretics that could not be stopped 3 months prior to and during the intervention.

### Intervention and randomization

In RENALIS, following baseline measurements, patients were randomized 1:1 (block size 4; performed by an independent pharmacist using computer-generated numbers) to receive once-daily linagliptin 5 mg or glimepiride 1 mg, each added to ongoing metformin (dose unchanged throughout the study) for 8 weeks. In RACELINES, participants were randomized 1:1:1 (block size 6) to double-blind treatment on top of metformin: (i) empagliflozin 10 mg daily (QD) for 8 weeks, then linagliptin 5 mg QD added for 8 weeks; (ii) linagliptin 5 mg QD for 8 weeks, then empagliflozin 10 mg QD added for 8 weeks; or (iii) gliclazide 30 mg QD for 8 weeks, then gliclazide 30 mg twice a day for 8 weeks. Only data from the first 8-week monotherapy phase were included in this pooled analysis. The combined dataset includes 71 patients, randomized to empagliflozin 10 mg (*N* = 20; RACELINES only), linagliptin 5 mg (*N* = 27; both trials) or a sulfonylurea [glimepiride 1 mg (RENALIS), gliclazide 30 mg (RACELINES); *N* = 24 total, *N* = 4 glimepiride, *N* = 20 gliclazide]. Patients were instructed to take their study drug daily in the evening at 8PM. The investigational products were over-encapsulated into visually identical capsules by independent Good Manufacturing Practice–certified organizations (RENALIS: ACE-Pharmaceutical, Zeewolde; RACELINES: Apotheek A15, Gorinchem, The Netherlands) without affecting pharmacokinetics or pharmacodynamics; patients and investigators remained blinded to treatment status until database unlock.

### Study protocol

The protocol for assessing renal physiological endpoints in fasting and postprandial states has been described previously [13–15], and is detailed in Supplementary data, S1. Briefly, participants adhered to standardized diets (sodium 9–12 g/day; protein 1.5–2.0 g/kg/day) and refrained from alcohol and vigorous physical activity for ≥24 h, and from nicotine and caffeine for ≥12 h prior to testing.

Following an overnight fast and water loading (500 mL), fasting blood and urine were collected. Measured GFR (mGFR) and ERPF were determined by standard clearance techniques, based on timed urine sampling, using inulin (RENALIS) or iohexol (RACELINES), and para-aminohippurate (PAH), respectively. Due to a global discontinuation of PAH production, 22 of 61 RACELINES participants underwent iohexol clearance only. After a tracer bolus and a 90-min equilibration under continuous infusion, two consecutive 45-min urine collections were obtained in the fasting state. Participants then ingested a standardized high-protein liquid meal (compositions in Supplementary data, S1) to induce postprandial hyperfiltration. Postprandial clearance measurements were performed after a zero-point void (45 min post-meal in RENALIS; 60 min in RACELINES), followed by two further 45-min urine collections. Urine and plasma were analyzed for tracer concentrations and electrolytes, with hematocrit assessed at the midpoint of each phase. To maintain diuresis, participants consumed 10 mL/kg of water during equilibration and 200 mL/h thereafter. Standardized voiding instructions were provided to optimize bladder emptying. Blood pressure and heart rate were measured at baseline and during collection periods using automated oscillometry on the brachial artery of the nondominant arm.

### Calculation of renal endpoints

GFR and ERPF were determined from inulin/iohexol and PAH clearances, respectively, based on timed urine collections and averaged across the two consecutive postprandial periods. Renal blood flow (RBF) was calculated as ERPF/(1 − hematocrit), filtration fraction as GFR/ERPF and renal vascular resistance (RVR) as mean arterial pressure/RBF. Intrarenal hemodynamic parameters, including P_GLO_, and afferent (R_A_) and efferent (R_E_) arteriolar resistances, were estimated using the Gomez equations (Supplementary data, S1) [17, 18]. FE_Na_ was calculated with inulin/iohexol as the reference marker. All renal hemodynamic variables were adjusted for body surface area [19].

### Statistical analysis

No formal sample size calculation was performed for this analysis. Before unblinding, inulin/iohexol extraction ratios were evaluated, and urine collection periods exhibiting substantial errors—defined as an extraction ratio ≥1.5 SD from the mean or >20% deviation before and after treatment—were discarded from the analyses. In RENALIS, collection errors occurred in three participants (all randomized to linagliptin), and in RACELINES in 14 participants (6 empagliflozin, 3 linagliptin, 5 gliclazide). For these participants, fasting mGFR and ERPF were calculated using continuous-infusion plasma clearance. Postprandial renal hemodynamics were only derived from urinary clearances, as steady state could not be assumed with the continuous infusion method, resulting in 10 RENALIS participants being included in the current dataset. Because RFR is derived from the change in GFR measured before and after the meal by standard clearance methods, patients with collection errors were excluded from analyses of meal-induced kidney hemodynamic responses and correlation analyses, resulting in 56 patients being included in these assessments. Statistical analyses were conducted in the per-protocol population using SPSS 28.0 (IBM, Chicago, IL, USA). Within-group comparisons were performed using paired *t*-tests for normally distributed data and Wilcoxon signed-rank tests for non-normal data. Associations were explored with Pearson’s correlation analyses. Two-sided significance was defined at α = 0.05. Data are presented as mean ± SD or ± SEM, median (interquartile range) or *n* (%), unless otherwise specified.

## RESULTS

The baseline characteristics of the 71 patients were generally well balanced across the treatment groups in the pooled dataset (Supplementary data, S2).

**Figure 1:**
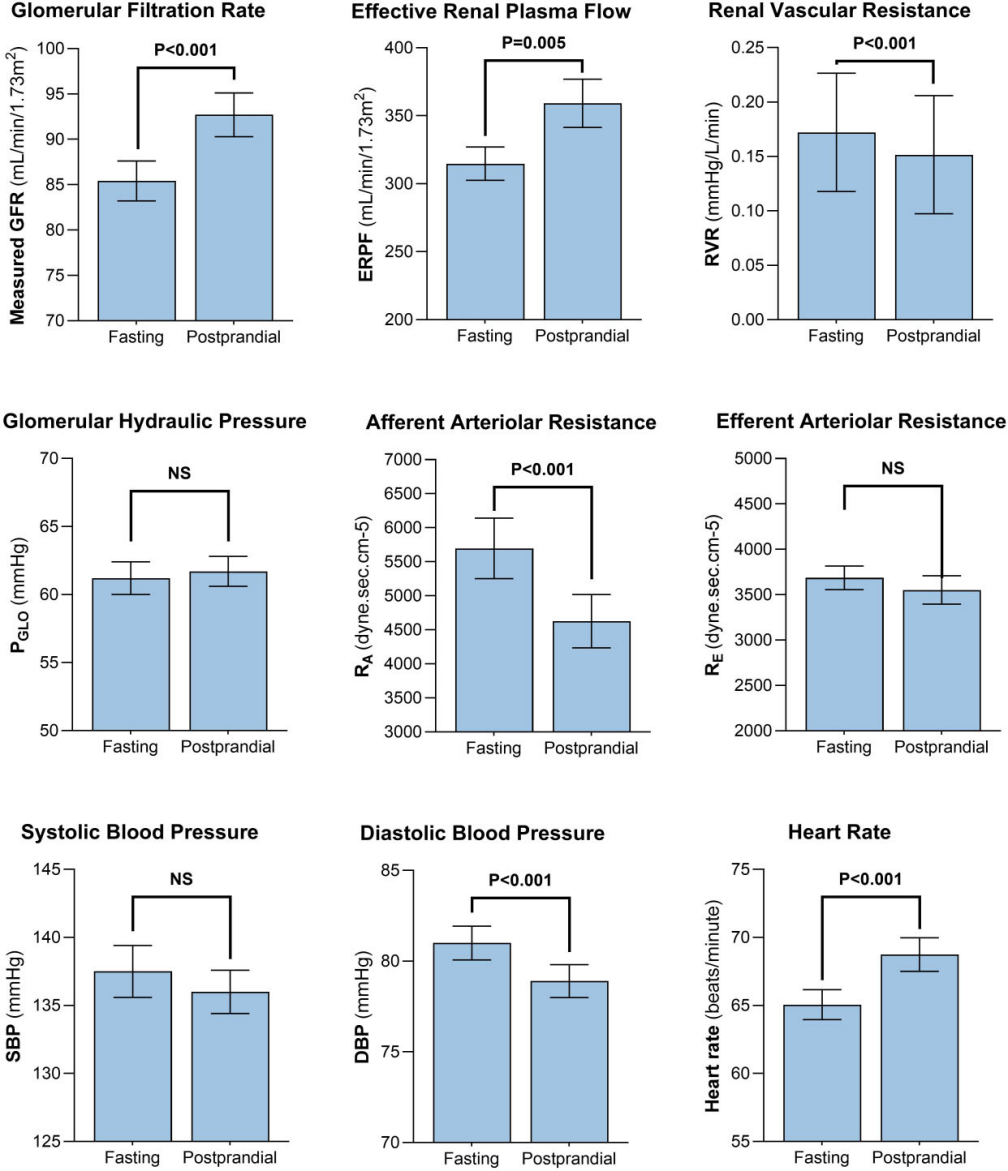
Effects of a high-protein liquid meal on renal hemodynamic functions and vital signs in T2D patients with preserved whole-kidney GFR (*N* = 56). Data are presented as mean ± SEM, and analyzed using a paired *T*-test. DBP, diastolic blood pressure; SBP, systolic blood pressure.

The meal increased mGFR (+7.3 ± 1.7 mL/min/1.73 m²) and ERPF (+44.3 ± 14.9 mL/min/1.73 m²), while reducing RVR (−0.02 ± 0.01 mmHg/L/min), R_A_ (−1068 ± 241 dyne/s/cm^5^; Fig. 1) and FE_Na_ (−0.21 ± 0.05; *P* < .001). The meal had no effect on systolic blood pressure, while diastolic blood pressure fell (−2.1 ± 0.5 mmHg; *P* < .001), and heart rate rose (+3.7 ± 0.8 beats/min; *P* < .001).

After 8 weeks, all treatments lowered HbA1c and fasting glucose (Table 1). Bodyweight increased with sulfonylurea (+1.0 ± 0.4 kg), was unchanged with linagliptin, and decreased with empagliflozin (−2.5 ± 0.5 kg). Empagliflozin reduced systolic (−9.5 ± 1.7 mmHg) and diastolic blood pressure (−4.7 ±1.0 mmHg), while linagliptin and sulfonylurea had no significant systemic hemodynamic effects.

**Table 1:** Effects of empagliflozin, linagliptin or sulfonylurea on anthropometric, metabolic and cardiorenal parameters after 8 weeks of treatment.

	**Empagliflozin 10 mg QD (*N* = 20)**			**Linagliptin 5 mg QD (*N* = 27)**			**Sulfonylurea^a^ (*N* = 24)**		
**Variables**	**Baseline**	**Week-8**	**Within-group *P*-value**	**Baseline**	**Week-8**	**Within-group *P*-value**	**Baseline**	**Week-8**	**Within-group *P*-value**
Metabolic variables									
Fasting plasma glucose, mmol/L	10.7 ± 0.5	8.6 ± 0.3	**<.001**	9.5 ± 0.5	8.4 ± 0.3	**.004**	10.0 ± 0.5	8.0 ± 0.4	**<.001**
HbA1c, %	7.8 ± 0.2	7.5 ± 0.3	**<.001**	7.5 ± 0.2	7.1 ± 0.2	**<.001**	8.1 ± 0.2	7.1 ± 0.2	**<.001**
HbA1c, mmol/mol	61.7 ± 2.0	58.6 ± 2.8	**<.001**	58.9 ± 1.9	54.3 ± 1.8	**<.001**	64.6 ± 2.7	54.7 ± 2.3	**<.001**
Body weight and composition									
Bodyweight, kg	100.3 ± 3.3	97.8 ± 3.2	**<.001**	101.0 ± 3.0	101.3 ± 3.0	.373	86.9 ± 2.6	87.9 ± 2.6	**.020**
BMI, kg/m^2^	31.5 ± 0.9	30.8 ± 0.9	**<.001**	31.3 ± 0.7	31.4 ± 0.7	.381	28.5 ± 0.6	28.9 ± 0.6	**.018**
Systemic hemodynamics									
SBP, mmHg	136 ± 3	127 ± 3	**<.001**	139 ± 4	135 ± 3	.082	138 ± 3	135 ± 3	.174
DBP, mmHg	81 ± 2	77 ± 1	**<.001**	81 ± 1	80 ± 2	.282	81 ± 2	81 ± 2	.533
Mean arterial pressure, mmHg	100 ± 2	94 ± 2	**<.001**	99 ± 2	99 ± 2	.174	100 ± 2	98 ± 2	*.095*
Heart rate, beats/minute	66 ± 2	63 ± 1	**.015**	63 ± 2	63 ± 2	.921	67 ± 2	67 ± 2	.782
Renal variables									
eGFR, mL/min/1.73 m^2^	79.7 ± 4.2	78.5 ± 4.0	.617	91.5 ± 2.0	89.1 ± 2.3	*.069*	88.1 ± 2.4	86.9 ± 2.2	.168
GFR, mL/min/1.73 m^2^	81.6 ± 5.0	73.3 ± 4.0	**.016**	91.9 ± 2.6	92.4 ± 3.1	.818	84.1 ± 3.3	81.3 ± 3.4	*.054*
ERPF, mL/min/1.73 m^2^	319 ± 25	289 ± 21	**.027**	349 ± 20	338 ± 19	.367	346 ± 31	343 ± 30	.875
FF, %	25.2 ± 1.0	25.0 ± 0.7	.860	27.3 ± 1.4	27.9 ± 1.3	.596	25.5 ± 1.3	25.1 ± 1.2	.823
RVR, mmHg/L/min	0.167 ± 0.020	0.169 ± 0.020	.890	0.153 ± 0.010	0.158 ± 0.010	.277	0.166 ± 0.014	0.172 ± 0.017	.416
P_GLO_, mmHg	59.2 ± 2.3	57.1 ± 2.1	*.064*	63.6 ± 1.4	63.5 ± 1.4	.926	58.9 ± 1.6	58.6 ± 1.3	.721
R_A_, dyne/s/cm^5^	5280 (3472–7836)	4111 (3552–9074)	.701	4023 (3155–6072)	4383 (3688–5520)	.455	5650 (3692–7366)	6507 (2892–8770)	.326
R_E_, dyne/s/cm^5^	3115 (2749–3428)	3091 (2644–3466)	.382	3852 (2943–4472)	3935 (3296–4525)	.247	3153 (2550–4461)	3264 (2498–4413)	.918
FENa, %	1.29 ± 0.13	1.40 ± 0.13	.762	1.13 ± 0.07	1.28 ± 0.09	**.018**	1.32 ± 0.09	1.46 ± 0.12	.298
UACR, mg/mmol	1.1 (0.4–4.3)	0.9 (0.4–2.8)	.184	1.2 (0.6–2.8)	1.2 (0.4–2.2)	.719	0.9 (0.4–1.7)	0.7 (0.4–2.5)	.274

Mean ± SEM or median (interquartile range). Paired *t*-tests or Wilcoxon signed rank tests were used for within-group comparisons. ^a^The sulfonylurea group included patients randomized to receive either glimepiride 1 mg QD (*N* = 4, in RENALIS) or gliclazide 30 mg QD (*N* = 20, in RACELINES). Values that are bold indicate statistical significance (*P* < .05), and italicized values indicate a statistical trend (*P* < .1). DBP, diastolic blood pressure; FF, filtration fraction; SBP, systolic blood pressure.

After 8 weeks, empagliflozin significantly reduced mGFR (−8.3 ± 3.1 mL/min/1.73 m²; *P* = .016) and lowered ERPF (−29.7 ±11.8 mL/min/1.73 m²; *P* = .027; Table 1). Sulfonylurea showed a trend toward increased mGFR (*P* = .054), with no change seen with linagliptin. No treatment significantly affected estimated intraglomerular hemodynamics. Linagliptin modestly increased FE_Na_ (+0.17; *P* = .018), with no effect observed with empagliflozin or sulfonylureas.

**Figure 2:**
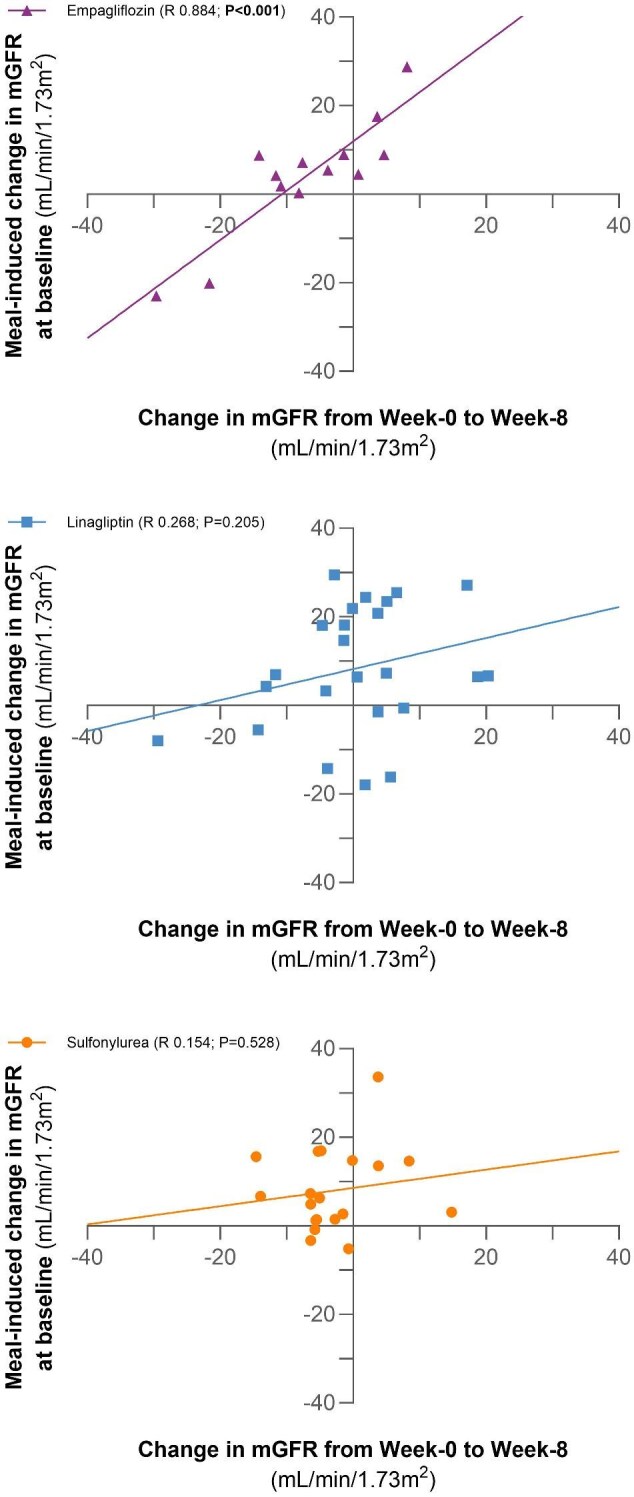
Correlation between the meal induced change in fasting to postprandial GFR and change in GFR from Week 0 to Week 8 with empagliflozin (*N* = 13), linagliptin (*N* = 24) and sulfonylurea (*N* = 19). Fewer patients were available for this analysis compared with the long-term fasting data due to urine collection errors in 3 patients in RENALIS (all randomized to linagliptin) and 14 patients in RACLINES (6 to empagliflozin, 3 to linagliptin and 5 to gliclazide). In these patients, fasting mGFR and ERPF were assessed using continuous infusion plasma clearance, while postprandial values were excluded because steady-state conditions could not be assumed with this clearance method.

Postprandial mGFR changes did not correlate with baseline mGFR or urinary albumin–creatinine ratio (UACR) (data not shown). Baseline postprandial mGFR changes strongly correlated with 8-week treatment-induced mGFR changes with empagliflozin (r: 0.884; *P* < .001), but not with linagliptin or sulfonylurea (Fig. 2).

## DISCUSSION

This pooled analysis of RENALIS and RACELINES provides further insights into the dynamic regulation of kidney hemodynamics in T2D, particularly in relation to RFR and the effects of commonly used glucose-lowering agents. Our findings reinforce that empagliflozin, but not linagliptin or sulfonylureas, reduces glomerular hyperfiltration in T2D patients. Importantly, among patients with preserved whole-kidney GFR (mean 86.5 mL/min/1.73 m^2^), those with reduced postprandially assessed RFR experienced the most pronounced acute GFR dip in response to empagliflozin; an effect associated with long-term kidney benefit [8–10]. These exploratory, hypothesis-generating findings suggest that RFR may offer additional, dynamic information to help identify individuals with subclinical hyperfiltration (i.e. a reduced RFR with preserved whole-kidney GFR), potentially complementing and refining simpler conventional biomarkers/predictors of therapeutic benefit, such as albuminuria, in identifying those who might derive the greatest physiological benefit from SGLT2 inhibitors.

We demonstrate that a high-protein meal increases mGFR and ERPF, accompanied by a reduction in RVR. Our data suggest that these postprandial changes are driven by enhanced proximal tubular Na reabsorption, evidenced by a meal-induced decrease in FE_Na_, which in turn may trigger a TGF response, resulting in the observed R_A_ reduction. The observed meal-induced changes in kidney hemodynamic are consistent with the recruitment of RFR and suggest that dynamic testing may unmask subclinical single-nephron hyperfiltration not evident from fasting GFR measurements alone [1]. Notably, postprandial changes in mGFR did not correlate with fasting mGFR or UACR, suggesting that traditional static measures may not always adequately capture kidney capacity/stress. Our findings emphasize the value of dynamic kidney tests in detecting relevant subclinical alterations beyond baseline data.

Our study advances insight into how glucose-lowering drugs affect kidney hemodynamics in T2D. Empagliflozin led to a significant mGFR reduction after 8 weeks, consistent with previous studies reporting initial, reversible GFR decline following SGLT2 inhibition [6, 7, 15]. This likely reflects a kidney hemodynamic reset through restored TGF [1]. Additional contributing mechanisms include reductions in bodyweight and systolic blood pressure (–2.5 kg and –9.5 mmHg with empagliflozin in our trial, respectively), and increased intratubular volume [1]. In contrast, linagliptin and sulfonylureas showed no clinically relevant effects on mGFR, ERPF or RVR, confirming that these agents primarily exert glucose-lowering effects without drug-specific kidney hemodynamic impact.

A key novel finding of this study is the strong correlation between postprandial mGFR change (i.e. RFR) and the mGFR reduction after 8 weeks of empagliflozin. This suggests that RFR may have potential as a biomarker to identify patients with both subclinical hyperfiltration and single-nephron hyperfiltration who are most likely to benefit kidney hemodynamically—and potentially long-term—from SGLT2 inhibition. Notably, this association was not observed with linagliptin or sulfonylureas, reinforcing a drug-specific, glucose-independent interaction between baseline renal hemodynamic status and mechanism-of-action of SGLT2 inhibitors. These findings are consistent with the concept of single-nephron hyperfiltration, whereby surviving nephrons adaptively increase GFR in response to nephron loss or hyperglycemia-induced tubular (hyper)reabsorption of sodium in the proximal tubule. However, such (compensatory) hyperfiltration is maladaptive over time, leading to glomerular injury, proteinuria and eventual further nephron-loss. By targeting TGF, SGLT2 inhibitors may interrupt this cycle early, particularly in those with diminished RFR indicative of a kidney operating at near-maximal filtration capacity.

Our observations are hypothesis-generating and may have potential clinical implications. If validated in larger, longer term studies, the assessment of RFR could complement existing measures such as eGFR and albuminuria for risk stratification in early DKD. A standardized postprandial or amino acid/dopamine-induced RFR test—preferably suitable for routine clinical implementation, for instance using cystatin C–based methods [20] or, pending further validation, emerging imaging modalities—might help clinicians to identify patients with subclinical hyperfiltration who are most likely to benefit from treatments that attenuate hyperfiltration. While SGLT2 inhibitors mark a clinical advance and have broad therapeutic indications, further work remains essential to better distinguish which patients are most likely to derive hemodynamic and renal benefit. Ultimately, such insights could inform a more targeted, personalized treatment approach to optimize cardiorenal protection while minimizing unnecessary exposure and potential harms.

Our study has several limitations. First, despite pooling data, the sample size remains relatively small, potentially introducing heterogeneity and phase error. Although we sought to minimize this by using homogeneous groups and standardized pre-study preparations, some heterogeneity is unavoidable. The predominantly elderly population and minor imbalances in gender, comorbidities and co-medications also affect generalizability. Second, in some patients, urine collection errors required continuous infusion methods for fasting kidney hemodynamics, limiting RFR assessment and excluding them from RFR analyses. As RFR testing evolves, standardized reliable protocols for both research and clinical use will be important. In this study, RFR was measured following a protein meal; it remains to be determined whether amino acid/dopamine infusions would produce similar results. Finally, estimations of intrarenal hemodynamic function using Gomez equations rely on assumptions [17, 18] not validated in the postprandial state; these results should therefore be interpreted cautiously.

In conclusion, this exploratory study suggests that dynamic GFR testing may help uncover subclinical hyperfiltration and could potentially aid in identifying T2D patients who are most likely to exhibit favorable renal responses to SGLT2 inhibition. The observed association between RFR and the treatment-response to empagliflozin may indicate an underlying pathophysiological link between preexisting glomerular stress and the renoprotective potential of this drug class. These findings are promising but, given the hypothesis-generating nature of our study, should be interpreted with caution. Conformation and validation in larger dedicated studies are warranted to further evaluate RFR as a potential predictive tool, alongside conventional biomarkers, to guide personalized therapy.

## Supplementary Material

gfag025_Supplemental_File

## Data Availability

The datasets generated and analyzed during this study are available from the corresponding author upon reasonable request. Data sharing will be subject to applicable institutional and ethical approvals and in accordance with participant confidentiality and relevant data protection regulations.
