# Supplementary material for: Renal functional reserve predicts GFR response to empagliflozin in RENALIS and RACELINES clinical trials
Source: Nephrol Dial Transplant. 2026 Feb 10;41(8):1520–8. doi: 10.1093/ndt/gfag025 (PMC13423819; doi:10.1093/ndt/gfag025)
Supplement: gfag025_Supplemental_File [file gfag025_supplemental_file.docx]

**SUPPLEMENTAL MATERIAL**

**Supplementary-S1: Materials and Methods**

Study Protocol

In the week preceding renal testing, participants followed standardized diets to reduce physiological variability: sodium intake of 9–12 g/day and protein intake of 1.5–2.0 g/kg/day. Additionally, participants were instructed to avoid vigorous physical activity and alcohol for at least 24 hours, and to abstain from nicotine and caffeine-containing products for at least 12 hours prior to testing. In RACELINES only, participants collected a 24-hour urine sample the day before each visit to assess albumin, creatinine, sodium, and glucose excretion.

After an overnight fast, participants consumed 500 mL of tap water to stimulate diuresis before arriving at the clinical research unit. Upon arrival, fasting blood and urine samples were collected. mGFR and ERPF were then measured using standard clearance techniques with timed urine collection: inulin in RENALIS (Inutest®, Fresenius-Kabi Austria GmbH, Graz, Austria) and iohexol in RACELINES (Omnipaque™, GE Healthcare B.V., Eindhoven, the Netherlands), alongside para-aminohippurate (PAH). In RENALIS, PAH was initially administered as aminohippurate sodium 20% (Merck Sharp & Dohme International, Whitehouse Station, NJ), but due to product discontinuation, this was later replaced in both the remainder of RENALIS and all of RACELINES by 4-Aminohippuric Acid Solution-20% (BaSodium 4-amino hippurate 20%, Basic Pharma, Geleen, the Netherlands). After a 10-minute bolus infusion of inulin (45 mg/kg) or iohexol (36 mg/kg), and PAH (3 mg/kg in RACELINES; 6 mg/kg in RENALIS), a continuous infusion of inulin or iohexol and PAH was administered at lower rates: 1350 mg/h (RENALIS) and 906 mg/h (RACELINES) for inulin/iohexol, and 762 mg/h (RENALIS) and 320 mg/h (RACELINES) for PAH. Due to a global PAH production halt, PAH became unavailable at the CRU during RACELINES, resulting in 22 of 61 participants being tested with iohexol only. After 90 min of equilibration, participants emptied their bladders to establish a required zero-point for fasting clearance measurements. Urine was collected by spontaneous voiding over two consecutive 45-minute periods. In RACELINES, participants subsequently consumed a high-protein (52 g) liquid meal (consisting of 15 g Nutricia® Protifar Plus, 30 mL PROSOURCE® Plus, 250 mL semi-skimmed milk, and a cup of marmalade) within 10 minutes to stimulate incretin hormone release and trigger postprandial hyperfiltration. During this period, iohexol/PAH infusion was maintained. In RENALIS, a pre-specified subgroup of 26 patients received a 400 mL liquid meal (Nutridrink Yoghurt Style®, Nutricia), with continued infusion of inulin/PAH throughout. Meal composition details for both trials are provided below.

Forty-five minutes after the meal in RENALIS, and one hour after the meal in RACELINES, participants again emptied their bladders to establish the postprandial zero-point, followed by two additional 45-minute urine collection periods. Urine samples from each collection were analyzed for inulin, iohexol, PAH, and electrolytes. Venous blood samples were drawn before and after each urine collection to measure the same analytes. Hematocrit was assessed at the midpoint of both fasting and postprandial urine collection periods. To promote diuresis, participants drank 10 mL/kg of tap water during the initial 90-minute equilibration, followed by a standardized intake of 200 mL/h for the remainder of the study day. Participants were encouraged to sit while voiding and to use a double-voiding technique to ensure complete bladder emptying.

Systolic blood pressure (SBP), diastolic blood pressure (DBP), mean arterial pressure (MAP), and heart rate (HR) were measured on arrival and during urine collection periods using an automated oscillometric device (Dinamap©, GE Healthcare, Little Chalfont, UK) on the brachial artery of the nondominant arm.

Total composition of the standardized liquid meals in RENALIS and RACELINES

|  | **RENALIS** | **RACELINES** |
| --- | --- | --- |
| **Energy**, kCal | 600.0 | 422.9 |
| **Protein**, grams | 23.6 | 52.0 |
| **Carbohydrate**, grams | 74.8 | 43.5 |
| **Fat**, grams | 5.8 | 4.1 |

Calculation of kidney hemodynamics

Kidney hemodynamics were estimated according to the model originally described by Gomez et al. Filtration pressure across the glomerular capillaries (ΔP_F_) is calculated by the following Gomez-formula, with the gross filtration coefficient (K_FG_) assumed to be 0.0554 mL/sec/mmHg (given a normal kidney physiology where GFR is 83.4 mL/min, i.e. mean of the current population), P_GLO_ is 60 mmHg (given Winton’s indirect estimates in the dog that glomerular pressure is roughly two-thirds of MAP), and normal glomerular oncotic pressure (πG) is 25 mmHg:

ΔP_F_ = GFR (mL/sec)/ K_FG_

πG (mmHg) is obtained from CM (plasma protein concentration within the glomerular capillaries), and calculated from TP (total protein concentration; g/dL) and FF:

CM = TP/FF ∙ Ln (1/1 – FF)

πG = 5 ∙ (CM – 2)

P_GLO_ was calculated by using above calculated variables and given the assumption that hydrostatic pressure in Bowman’s space (P_BOW_) was 10 mmHg, as follows:

P_GLO_ = ΔP_F_ + P_BOW_ + πG

P_GLO_ = (GFR/ K_FG_) + 10 mmHg + [5*(TP/FF*Ln(1/ –FF)−2)]

Finally, in order to calculate renal vascular resistance of the afferent (R_A_) and efferent (R_E_) renal arteriole, we used the principles of Ohm’s law, and the factor 1328 to convert to dyne∙sec∙cm^-5^:

R_A_ = [(MAP–P_GLO_/RBF] ∙ 1328

R_E_ = [GFR/(K_FG_*(RBF–GFR)] ∙ 1328

**Supplementary-S2:** Demographic and baseline clinical characteristics

| **Variables** | **Empagliflozin**  (N=20) | **Linagliptin**  (N=27) | **Sulfonylurea**  (N=24) |
| --- | --- | --- | --- |
| Age, *years* | 65.7 ±6.3 | 64.3 ±6.9 | 65.6 ±7.4 |
| Male, *n* (%) | 16 (80) | 24 (88.9) | 19 (79.2) |
| Diabetes duration, *years* | 10.8 ±6.6 | 9.5 ±5.7 | 8.7 ±5.5 |
| RAS inhibitor use, *n (%)* | 14 (70.0) | 21 (77.8) | 12 (50.0) |
| Bodyweight, *kg* | 100.3 ±14.9 | 101.0 ±15.4 | 86.9 ±12.7 |
| Body mass index, *kg/m^2^* | 31.5 ±4.2 | 31.3 ±3.7 | 28.5 ±3.0 |
| Systolic blood pressure, *mmHg* | 136 ±3 | 139 ±4 | 138 ±3 |
| Diastolic blood pressure, *mmHg* | 81 ±2 | 81 ±1 | 81 ±2 |
| Mean arterial pressure, *mmHg* | 100 ±2 | 99 ±2 | 100 ±2 |
| Heart Rate, *beats/minute* | 66 ±2 | 63 ±2 | 67 ±2 |
| HbA1c, *%* | 7.8 ±0.8 | 7.5 ±0.9 | 8.1 ±1.2 |
| HbA1c, mmol/mol |  |  |  |
| Fasting plasma glucose, *mmol/L* | 10.7 ±0.5 | 9.5 ±0.5 | 10.0 ±0.5 |
| Fasting mGFR, *mL/min/1.73m^2^* | 81.6 ±5.0 | 91.9 ±2.6 | 84.1 ±3.3 |
| Albumin-creatinine ratio, *mg/mmol* | 1. 1 [0.4-4.3] | 1.2 [0.6-2.8] | 0.9 [0.4-1.7] |
| Microalbuminuria*, *n (%)* | 5 (25.0) | 6 (22.2) | 3 (12.5) |

Data are shown as mean ±SD/SEM, median [IQR] or n (%).*Defined as a urinary albumin-creatinine ratio ≥3 mg/mmol. Abbreviations: mGFR, inulin/iohexol-measured glomerular filtration rate; HbA1c, glycated hemoglobin; RAS, renin-angiotensin-system.
